# Voltage-Controlled Magnetoresistance in Silicon Nanowire Transistors

**DOI:** 10.1038/s41598-018-33673-8

**Published:** 2018-10-12

**Authors:** Yawen Zhang, Jiewen Fan, Qianqian Huang, Jiadi Zhu, Yang Zhao, Ming Li, Yanqing Wu, Ru Huang

**Affiliations:** 1 Key Laboratory of Microelectronic Devices and Circuits (MOE), Institute of Microelectronics, Peking University, Beijing, 100871 China; 2 National Key Laboratory of Science and Technology on Micro/Nano Fabrication, Beijing, 100871 China; 3 Wuhan National High Magnetic Field Center and School of Optical and Electronic Information, Huazhong University of Science and Technology, Wuhan, 430074 China

## Abstract

Magneto-electronic logic is an innovative approach to performing high-efficiency computations. Additionally, the ultra-large scale integration requirement for computation strongly suggests exploiting magnetoresistance effects in non-magnetic semiconductor materials. Here, we demonstrate the magnetoresistance effect in a silicon nanowire field effect transistor (SNWT) fabricated by complementary metal-oxide-semiconductor (CMOS)-compatible technology. Our experimental results show that the sign and the magnitude of the magnetoresistance in SNWTs can be effectively controlled by the drain-source voltage and the gate-source voltage, respectively, playing the role of a multi-terminal tunable magnetoresistance device. Various current models are established and in good agreement with the experimental results that describe the impact of electrical voltage and magnetic field on magnetoresistance, which provides design feasibility for the high-density magneto-electronic circuit. Such findings will further pave the way for nanoscale silicon-based magneto-electronics logic devices and show a possible path beyond the developmental limits of CMOS logic.

## Introduction

Magneto-electronics have utilized the properties of spin and charge to develop new multi-functional devices for applications in magnetic sensors^1^, non-volatile memory^2–4^ and magneto-electronic logic devices^5–10^. Magneto-electronic devices are also expected to go beyond the performance limits of complementary metal-oxide-semiconductor (CMOS) devices^11,12^. Hence, magnetic logic devices have attracted significant attention recently, with most investigations based on spintronic devices^5–7,13,14^ with spin-dependent transport. Although spintronic devices do have superiority in terms of ultra-low power consumption, the fabrication process of spintronic devices is relatively complicated and incompatible with current CMOS technologies.

Recently, magnetoresistance (MR) effects in silicon-based devices have been found in high electric fields, showing strong inhomogeneous magnetoresistive (IMR) effects^15–18^. Benefiting from the excellent compatibility with the mainstream CMOS logic technologies and the relatively long spin lifetime of the silicon material, magnetoresistive effects in silicon-based devices have attracted growing attention. For extremely high-density integration, nanoscale silicon devices with the capability of magneto control are urgently required, with only a few works having been demonstrated. Additionally, the magnetoresistance of reported two-terminal devices^15^ cannot be effectively controlled once the doping profile and carrier injections are fixed. Therefore, multi-terminal devices, such as transistors, might be preferred in magneto-electronic logic to achieve voltage control and could be helpful for clarifying the dynamic process of magnetoresistance change in semiconductor materials.

Here, we report the magnetoresistive effect in a silicon nanowire transistor, which is considered to be one of the most promising candidates for an advanced technology node with an extremely high integration density^19^. It is determined that the sign and the magnitude of the magnetoresistance can be effectively controlled by the drain voltage and the gate voltage in silicon nanowire transistors, respectively. The current model, which is in good agreement with experimental results, is introduced to describe the impacts of the electrical voltage and magnetic field on magnetoresistance. According to the magneto-electronic device in our work, the findings will pave the way for silicon-based magneto-electronic logic on the nanoscale, showing promise beyond the developmental limits of CMOS logic.

## Results

**Figure 1:**
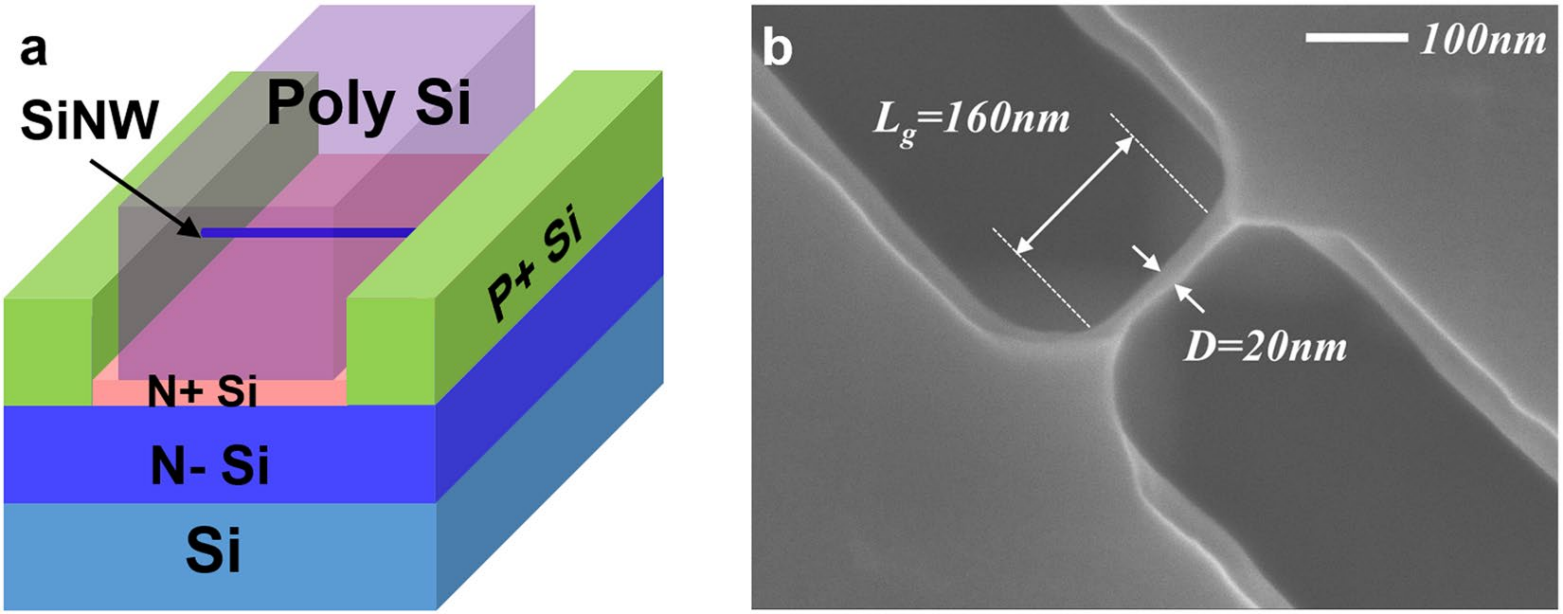
Structure of gate-all-around silicon nanowire transistors. (**a**) Schematic of the p-type gate-all-around silicon nanowire transistor. A silicon nanowire is suspended from the substrate by wet etching after silicon fin patterning. (**b**) Top view SEM image of the p-type silicon nanowire. The diameter of the nanowire and the gate length are directly defined by electron beam lithography. The diameter and the gate length of the silicon nanowire transistor are 20 nm and 160 nm, respectively.

The device used in our study is a p-type gate-all-around silicon nanowire transistor, whose schematic diagram is shown in Fig. 1a. A silicon nanowire is suspended from the substrate via wet etching after silicon fin patterning (see Supplementary Fig. S1). The diameter (D) of the nanowire and the gate length (L_g_) are directly defined by the electron beam lithography. The silicon nanowire structure is also observed by scanning electron microscopy (SEM). Figure 1b shows the SEM image of the nanowire structure with a typical diameter of ~20 nm and a gate length of 160 nm. The channel is lightly doped to obtain high carrier mobility, while the substrate below the nanowire is heavily doped to suppress the parasitic bottom transistors through the threshold voltage (V_th_) mismatch. In addition, a large volume of silicon remains in the source/drain (S/D) region, which can substantially reduce the parasitic resistance and improve the driving current (I_ds_). The transfer characteristics of this p-type transistor are shown in Supplementary Fig. S2, demonstrating the large on-off current ratio and the typical subthreshold slope dependence on the temperature.

### Drain Control of the Magnetoresistance Sign

**Figure 2:**
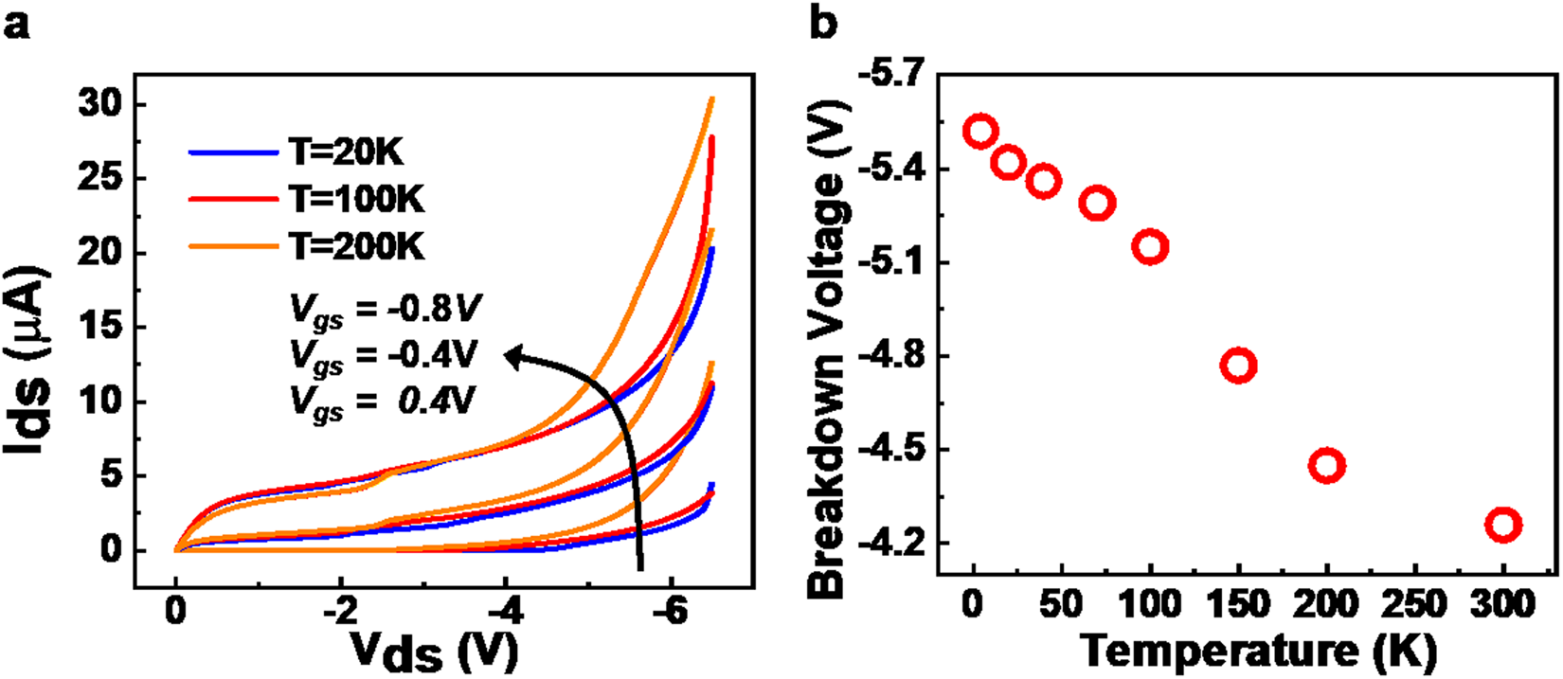
Output characteristics of silicon nanowire transistors at various temperatures. (**a**) Drain-source current (I_ds_) vs. drain-source voltage (V_ds_) characteristics of the device with V_gs_ increasing from −0.8 V to 0.4 V. (**b**) Breakdown voltage decreases as temperatures increase from 4.3 K to 300 K at V_gs_ = 0.4 V. Breakdown voltage is defined as the voltage at which the slope of the output curve reaches 1.0 × 10^−5^A/V.

Figure 2a shows the typical output characteristics of the silicon nanowire transistor at different temperatures. With the drain-source voltage (V_ds_) increasing, the electric field across the p-n junction at the drain increases until tunneling occurs in the carrier. When V_ds_ exceeds the breakdown voltage (extracted as the voltage where the slope of output curve is 1.0 × 10^−5^A/V), the drain current deviates from the current saturation and begins to increase nonlinearly. As the temperature increases from 4.3 K to 300 K, the kinetic energy of electrons gradually increases, leading to a decrease in the breakdown voltage, as shown in Fig. 2b. This negative temperature dependence further verifies that the breakdown mechanism is Zener tunneling rather than avalanche ionization.

**Figure 3:**
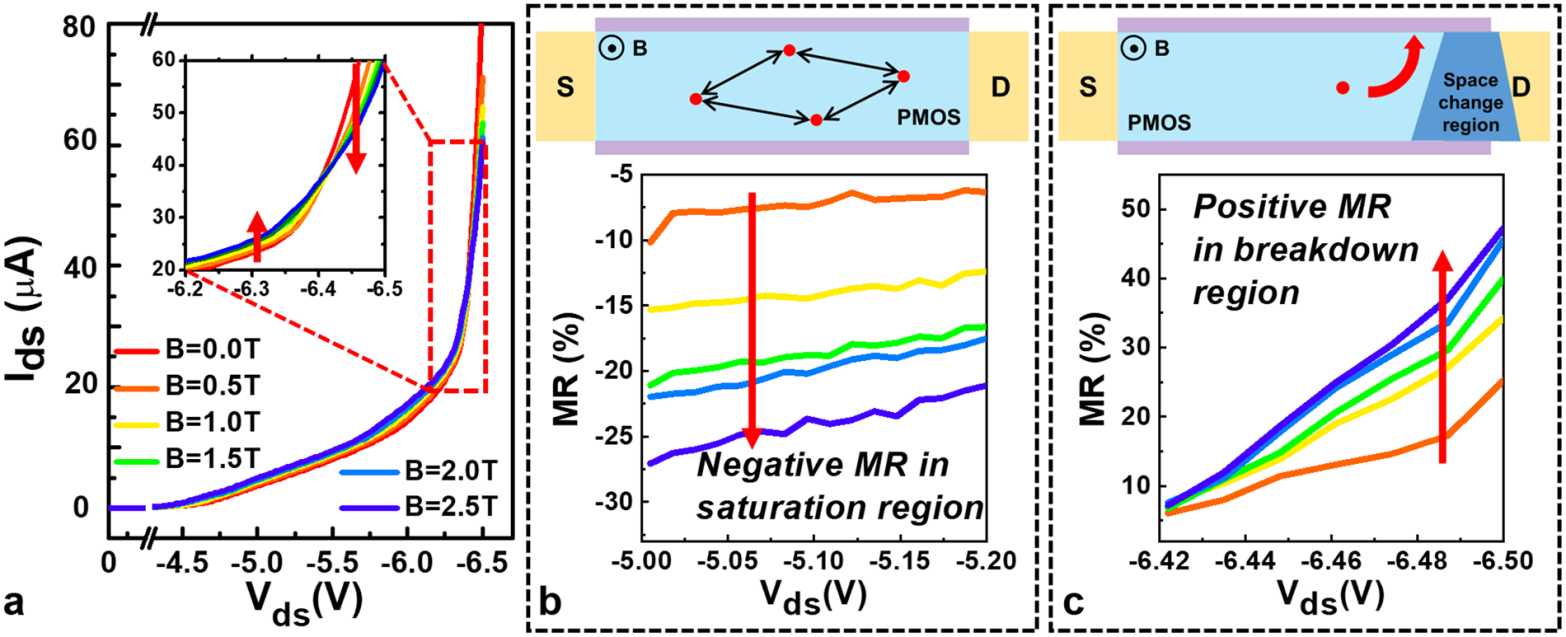
Drain-voltage-controlled sign of magnetoresistance. (**a**) I_ds_ as a function of V_ds_ with different magnetic fields in the off state of SNWT at 4.3 K. (inset) The sign of the MR changes as the drain voltage increases, demonstrating that the internal dynamic changes as the drain voltage increases. (**b**) Negative MR in the saturation region due to the suppression of a magnetic field on quantum interference effects. (**c**) Positive MR in the breakdown region is induced by an external magnetic field changing the space charge region.

In order to investigate the MR effects of SNWTs at different working states, the current-voltage (I–V) characteristics of SNWTs were measured at 4.3 K in various magnetic fields with V_ds_ changed from −5.0 V to −6.5 V, which is around the Zener breakdown voltage in silicon. It is shown that the drain current in the silicon nanowire can be effectively tuned by applying a perpendicular magnetic field as shown in Fig. 3. Here, the magnetoresistance is defined as the change ratio of the resistance with the presence of a magnetic field

$$MR=\frac{R(B)-R(0)}{R(0)}\times 100 \% ,$$

where R(B) and R(0) are the resistance $(R=\frac{{V}_{ds}}{{I}_{ds}})$ with and without applying magnetic fields and can be easily extracted from the drain-source current.

As shown in Fig. 3a, the output characteristics of nanowires show a distinct transition from the negative to positive magnetoresistance with V_ds_ increasing at 4.3 K. The magnetoresistance is negative in the saturation region and becomes positive due to effect of Zener breakdown. These results indicate that when a magnetic field is applied perpendicularly to SNWTs, the magnetoresistance sign can be controlled by the drain voltage due to different conductive mechanisms.

### A. Negative magnetoresistance

At low temperatures, the transport of silicon nanowire transistors in the saturation region is strongly influenced by impurity scattering with quantum corrections to the conductance. In a disordered electronic system, quantum interference could be significant, which would lead to the loss of conductivity at 0 K if the impurity scattering is irrelevant to spin^20^. Fig. 3b shows the measured negative magnetoresistance at 4.3 K with the drain voltage from −5.0 V to −6.0 V. This negative magnetoresistive effect is closely related to the suppression of the weak localization by applying a magnetic field^21^.

The weak localization comes from the quantum interference effect between self-crossing diffusion paths and their time reverse. As the lengths of two corresponding paths are the same, quantum phases can be compensated by one another, resulting in a decrease in the conductance, as shown in Fig. 3b. Since the self-crossing diffusion paths are more likely to be found in lower-dimensional systems, the weak localization effect is much stronger in our 1-D silicon nanowire transistors compared with planar devices or FinFETs.

If a magnetic field is applied perpendicularly to the silicon nanowire transistor, the magnetic field would destroy the coherent backward scattering between two corresponding diffusion paths. Specifically, the applied magnetic field would suppress the weak localization effect. The intensity of weak localization is weakened in the presence of a magnetic field, resulting in increased conductivity. Therefore, the applied magnetic field enhances the drain current by increasing the conductivity of the channel, which results in negative magnetoresistance. The detailed relationship is presented in Supplementary Note 1.

### B. Positive magnetoresistance

For silicon nanowire transistors, the space charge region at the drain is almost uniform without a magnetic field. The width of the space charge region at the drain is determined by both the diffusion process and the “built-in” electric field. However, the above equilibrium of spatial distribution would be disturbed by applying an external magnetic field. The magnetic field causes deflection of the electron and hole moving in the channel and to the drain due to the Lorenz force. Therefore, when a magnetic field is applied, the spatial distributions of the electron and hole concentrations would change. As shown in Fig. 3c, the space charge region presents a trapezoidal distribution^22^, and an inhomogeneous electric field^23^ can be observed in the space charge region.

When V_ds_ is small, the transport of the silicon nanowire transistor is less affected by this trapezoidal distribution in the space charge region at the drain. With V_ds_ increasing, the electric field inside the depletion region at the drain breaks off the covalent bonds of the electrons or holes, leading to Zener breakdown. The transport properties of the Zener breakdown strongly depend on the tunneling electric field and the carrier concentrations in the drain space charge region, which means that the magnetic field can distinctly influence Zener tunneling. The trapezoidal distribution in the space charge region will lead to an irregular Zener tunneling length along the diametrical direction of the SNWT drain junction. According to the WKB approximation^24^, for a wider depletion region, the tunneling current is smaller due to the larger Zener tunneling length. In addition, for a narrower depletion region, it is difficult for carriers to Zener tunnel due to the strong inhomogeneous magnetoresistive^23^ effect. As a result, the total tunneling resistance of the silicon nanowire transistor will increase when applying a magnetic field, which is consistent with the experimental observations. In addition, due to the nanowire structure of our devices, when carriers are deflected by a Lorentz force, they will suffer more impacts from surface roughness scattering. This phenomenon also leads to a decrease in the kinetic energy of the carrier and thus a subsequent decrease in the positive magnetoresistance.

### Gate Control of the Magnetoresistance Magnitude

**Figure 4:**
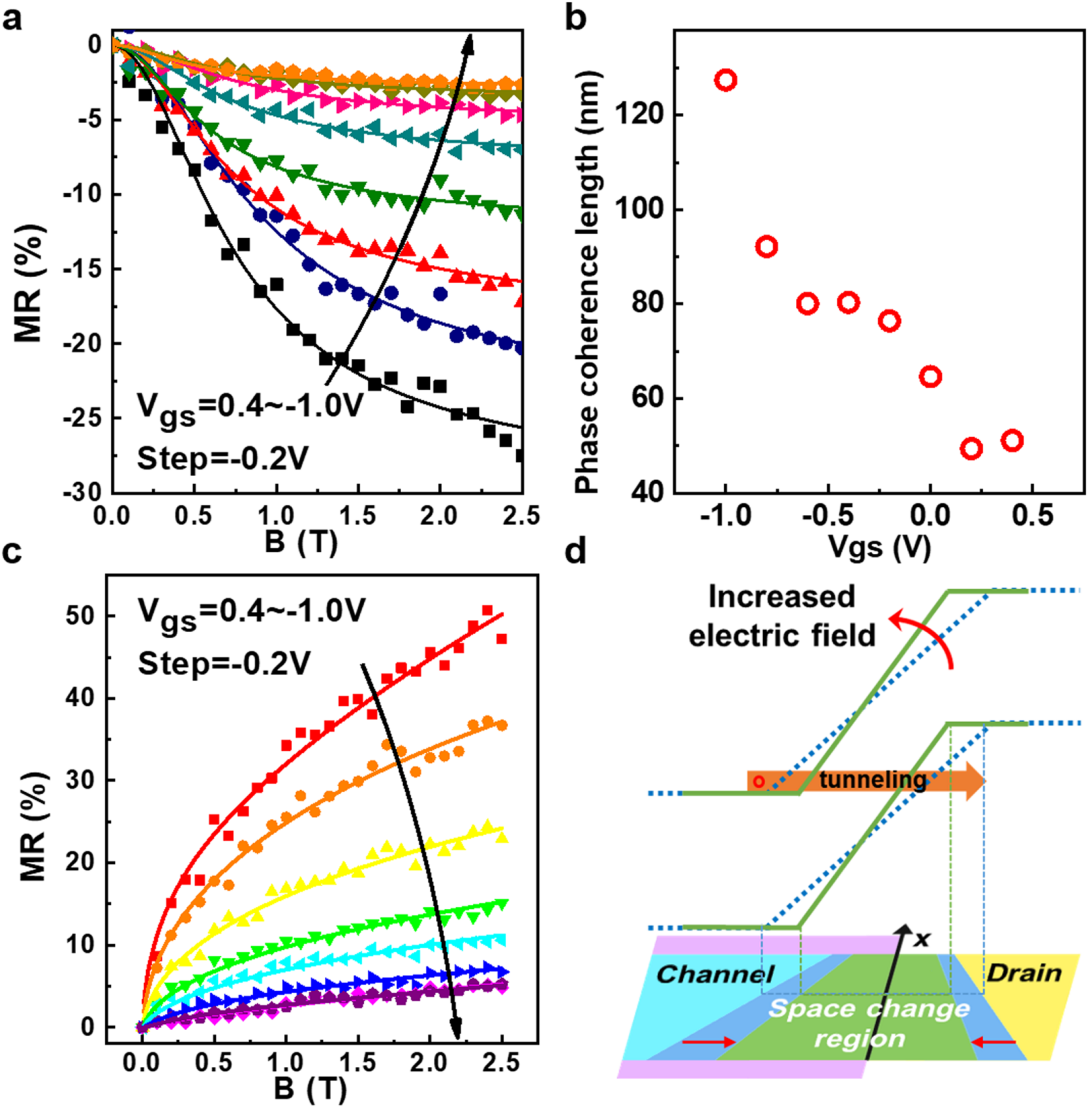
Gate-voltage-controlled magnitude of magnetoresistance. (**a**) Negative MR as a function of the magnetic field at different V_gs_ at V_ds_ = −5 V and Temp. = 4.3 K. Solid lines show the fitting results using the 1D weak localization model. (**b**) The phase coherence lengths at different gate voltages are calculated. It increases with a decreased gate voltage due to an increase in the density of the carriers. (**c**) Positive MR as a function of magnetic field at different V_gs_ at V_ds_ = −6.5 V and Temp. = 4.3 K. (**d**) The schematic of a trapezoidal distribution in the space charge region driven by an external magnetic field and the WKB approximation of Zener tunneling. The effects of the gate voltage on the carrier concentration over the space charge region are directly related to the tunneling electric field.

Compared with two-terminal p-n junctions, an important feature of transistors is that the drain current can be tuned by the gate voltage. In order to further investigate the gate-voltage-controlled magnetoresistance, the magnetic-field dependence and the gate-voltage dependence of magnetoresistive effects were measured and extracted in the saturation region and the breakdown region, as shown in Fig. 4a,c respectively.

Figure 4a shows magnetoresistance as a function of the magnetic field and the gate voltage at V_ds_ = −5 V. The observed negative magnetoresistance shows an increasing trend from the saturation region towards the cut-off region, which can be attributed to an increase in carrier concentration driven by the gate voltage. In the cut-off region (V_gs_ = 0.4 V), the magnitude of the negative magnetoresistive effects can reach up to −28% in 2.5 T.

In order to precisely characterize the magnitude of the magnetoresistance adjusted by the gate voltage in the saturation region, we model the negative magnetoresistance using 1D weak localization correction^21^. Here, the crucial parameter is the electron phase coherence length *l*_*φ*_, which controls the strength of weak localization. In our disordered quasi-1D system, the increase in the carrier concentration makes the electron phase coherence length increase and thus weakens the phenomenon of the quantum coherence effect. The resistance with 1D weak localization using the Hikami-Larkin-Nagaoka (HLN) model^21^ is given by

$$\frac{{\rm{\Delta }}R}{R(B)R(0)}=\alpha [{l}_{\phi }-{(\frac{1}{{{l}_{\phi }}^{2}}+\frac{{e}^{2}{B}^{2}{W}^{2}}{3{\hslash }^{2}})}^{-0.5}]$$

where *α* is a fitting parameter $\hslash$and is the reduced Plank constant. Figure 4a displays the fitting results between the 1D weak localization model (solid lines) and the experimental results. It can be calculated that the extracted value of *l*_*φ*_ increases from 51 nm for V_gs_ = 0.4 V to 128 nm for V_gs_ = −1.0 V, as shown in Fig. 4b. The electron phase coherence length increases with the reduced gate voltage due to an increase in the density of the carriers, which is in good agreement with the reported results^25^. The theoretical and experimental results show that the negative magnitude of the magnetoresistance can be tuned by the gate voltage.

To further investigate the influence of the gate voltage on positive magnetoresistive effects, the magnetoresistance-magnetic field (MR-B) curves of SNWTs in the Zener breakdown region were measured for various values of V_gs_, as shown in Fig. 4c. Due to the increase in the carrier concentration in the channel, which was driven by the gate voltage, the observed magnetoresistance shows an increasing trend as V_gs_ decreases. As shown in Fig. 4d, the trapezoidal distribution of the space charge region becomes indistinct as the gate voltage increases, resulting in the reduction of positive magnetoresistive effects.

In order to quantitatively investigate the magnitude of the magnetoresistance adjusted by the gate voltage, we model the positive magnetoresistive effects using Zener tunneling approximation^26,27^. Since the spatial distribution of the space charge region induced by the magnetic field is nonuniform, here we assume that the space charge region presents a trapezoidal distribution $W={W}_{0}[a(x+b)+1]$, where *W*_0_ is the original width of the space charge region, *a* and *b* are fitting parameters and *x* is the location along an interface of the p-n junction, as shown in Fig. 4d. The effects of the gate voltage on the carrier concentration over the space charge region can be calculated through the transport equations. Taking the WKB approximation^24^ and the trapezoidal distribution of the space charge region into the Zener tunneling equations, the ratio of tunneling currents with a magnetic field to the original tunneling currents can be calculated as follows,

$$\frac{{I}_{B}}{{I}_{0}}=\frac{1}{L\cdot {A}_{1}\cdot {W}_{0}\cdot \mathrm{Exp}(C)}{Ei[\frac{C}{{W}_{0}}W({A}_{2},B)]|}_{0}^{L}$$

where *A*_1_ and *A*_2_ are the fitting parameters related to the carrier concentration, *Ei*(x) is the exponential integral function, L is the diameter of the silicon nanowire and C is a constant. The detailed derivation is presented in Supplementary Note 2. The calculated values by the model (solid lines) show good agreement with the experimental results, as shown in Fig. 4c. According to the results, the effects of magnetic fields on the spatial distribution of the space charge region become irrelevant with larger values of V_gs_ because the increase in carrier concentrations shortens the space charge region width, as shown in Fig. 4c. Conversely, as the gate voltage decreases, the positive magnetoresistance can reach 47% at 2.5 T when V_gs_ = 0.4 V. In light of this, the gate voltage can effectively control the spatial distribution of the space charge region via magnetic fields and, subsequently, the magnitude of the positive magnetoresistance effect.

## Discussion

To summarize, we have investigated the magnetoresistive effects in silicon nanowire transistors by applying a perpendicular magnetic field. In the saturation region, due to the suppression of a magnetic field on quantum interference effects, the negative magnetoresistance can reach −28% at 2.5 T. By contrast, in the breakdown region, the positive magnetoresistance originates from the change of the Zener tunneling current, which is induced by a change in the space charge region, and a 47% positive magnetoresistance is observed with a magnetic field of 2.5 T. The carrier concentration change induced by the gate voltage can greatly influence the magnitude of the magnetoresistance, which would open a pathway towards magnetoresistance enhancement. In addition, the mechanism of electrical voltage and magnetic field on the magnetoresistance of the device are quantitatively investigated, and a current model drawn from weak localization and trapezoidal distribution of the space charge region matches well with the experimental results. Our model will also be helpful for understanding the dynamic physical processes of the magnetoresistance change in SNWTs.

In this work, it is determined that not only can the sign of the magnetoresistance be tuned by the drain voltage, but the magnitude of the magnetoresistance can also be controlled by the gate voltage. Conventional magnetic logic devices usually require different magnetic fields applied to each device, which consumes significant energy to adjust the magnetic field. Since the sign and magnitude of magnetoresistance can be adjusted simultaneously by the voltages in the SNWT, only a uniform magnetic field needs to be applied on all the magnetoresistive devices for magnetic-electronic logic operation. Moreover, the silicon nanowire transistors used in this work show high compatibility with mainstream CMOS technology and high integration density. Therefore, the voltage-controlled magnetoresistive effects found in the SNWT in this work pave the way for magneto-electronics logic on the nanoscale and provide additional methods to surpass the developmental limits of CMOS logic performance.

## Methods

### Device fabrication

The device fabrication is performed based on the following steps. The patterns of the source, the drain and the fin bar connecting the source and the drain are defined by electron beam lithography. After the silicon fin is patterned, a silicon nanowire is suspended from the substrate using wet etching with tetramethylammonium hydroxide (TMAH). The suspended silicon fin bar connecting the source and the drain is reduced to the nanoscale by a sacrificial isolation to form the silicon nanowire. Ion implantation is performed to suppress the parasitic bottom transistor. A polysilicon gate line across the silicon nanowire is formed via electron beam lithography and subsequent etching, and then a gate-all-around structure is formed. The highly doped source and the drain are formed via ion implantation, followed by high-temperature annealing.

### Electrical measurements

All the electrical measurements were performed in a vacant environment. The tests were carried out using an Agilent B1500A semiconductor parameter analyzer.

## Electronic supplementary material

Supplementary Information

## Data Availability

All data supporting this study and its findings are available within the article, the Supplementary Information and associated files. Any source data deemed relevant is available from the corresponding author upon request.
