# Supplementary material for: Voltage-Controlled Magnetoresistance in Silicon Nanowire Transistors
Source: Sci Rep. 2018 Oct 12;8:15194. doi: 10.1038/s41598-018-33673-8 (PMC6185961; doi:10.1038/s41598-018-33673-8)
Supplement: Supplementary file 1 — Supplementary Information [file 41598_2018_33673_MOESM1_ESM.docx]

*Supplementary Information for*

**Voltage-Controlled Magnetoresistance in Silicon Nanowire Transistors**

Yawen Zhang^1^, Jiewen Fan^1^, Qianqian Huang^1,2*^, Jiadi Zhu^1^, Yang Zhao^1^, Ming Li^1,2^, Yanqing Wu^3^ and Ru Huang^1,2*^

^1^*Key Laboratory of Microelectronic Devices and Circuits (MOE), Institute of Microelectronics, Peking University, Beijing 100871, China.*

*^2^National Key Laboratory of Science and Technology on Micro/Nano Fabrication, Beijing 100871, China*

*^3^Wuhan National High Magnetic Field Center and School of Optical and Electronic Information, Huazhong University of Science and Technology, Wuhan 430074, China*

^*^Correspondence should be addressed to: ruhuang@pku.edu.cn, hqq@pku.edu.cn


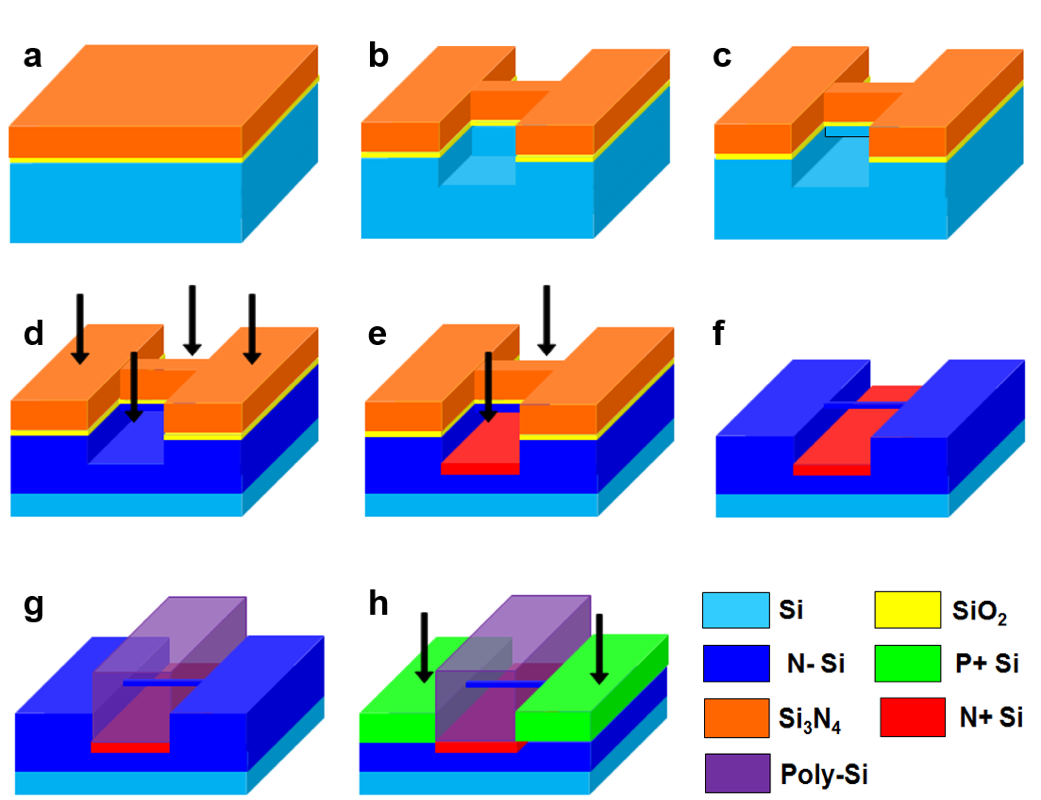


**Supplementary Figure 1 | Fabrication process of the silicon nanowire transistors. Our silicon nanowire transistor can be fabricated by the following steps.** **(a)** Defining the active area and depositing the hard masks; **(b)** Patterning the source, the drain and the fin bar regions by electron beam lithography and dry etching; **(c)** Anisotropic wet etching the silicon substrate with an tetramethylammonium hydroxide (TMAH) solution, and forming the suspended silicon fin bar connecting the source and the drain; **(d)** Performing the high-energy phosphorus ion implantation in the active region to form N-doped substrate; **(e)** Performing the low-energy phosphorus ion implantation in the active region to suppress the parasitic bottom transistor; **(f)** Removing the hard masks of silicon oxide and the silicon nitride; **(g)** Patterning the polysilicon gate line across the silicon nanowire by electron beam lithography and subsequently etching to form a gate-all-around structure; **(h)** Forming the source and the drain regions by boron ion implanting and high-temperature annealing.


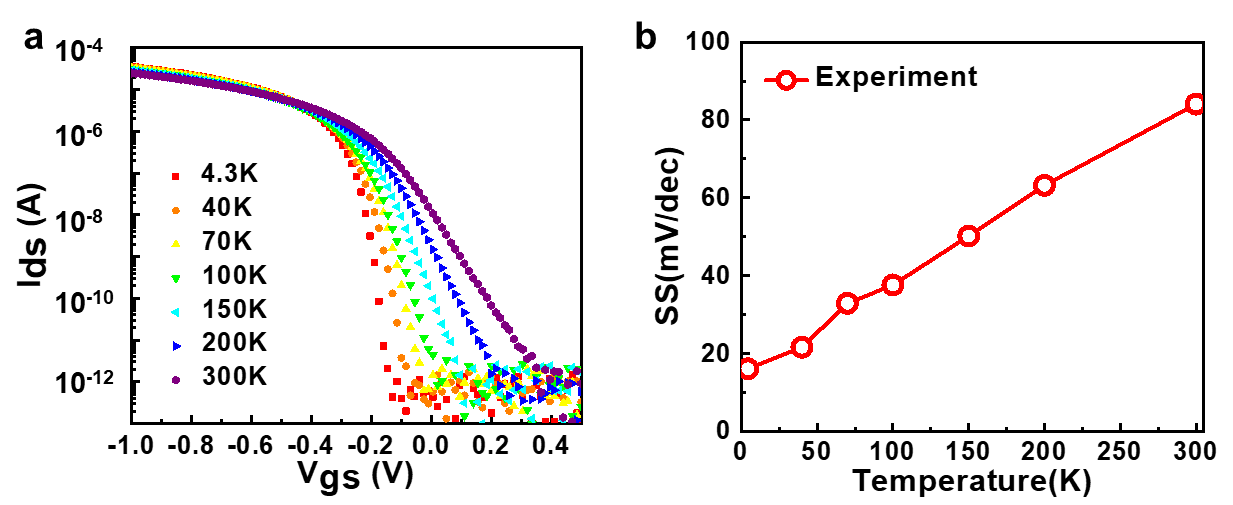


**Supplementary Figure 2 | Transfer characteristics of the silicon nanowire. (a)** The measured transfer characteristics of the fabricated SNWT under various temperatures from 4.3 K to 300 K. The on-state drain current slightly increases from 300 K to 4.3 K mainly because of the reduction of the phonon scattering with a decreasing temperature. **(b)** The subthreshold slope (SS) changes from 84 mV/decade at 300 K to 16 mV/decade at 4.3 K.

**Supplementary Note 1. Negative magnetoresistance in silicon nanowire transistors based on weak localization.**

The change of the conductivity induced by the magnetic field in silicon nanowire transistors is given by ^S1^

$$\Delta\sigma(B)=\frac{\alpha e^{2}}{2\pi^{2}\hbar}\left[ \Psi\left( \frac{1}{2}+\frac{\hbar}{4eBD\tau_{\varphi}} \right)+ln \left( \frac{4eBD\tau_{\varphi}}{\hbar} \right) \right],$$

where $\alpha$ is the weak localization coefficient, *Ψ* is the Digamma function, $\hbar$ is the reduced Plank constant, *D* the diffusion coefficient and $\tau_{\varphi}$ is the phase coherence time. The expression of the drain current in the saturation region is $I_{ds}=\frac{1}{2}\frac{W}{L}\mu_{eff}C_{ox}\left( V_{gs}-V_{th} \right)^{2}$, where *W/L* is the aspect ratio, $\mu_{eff}=\frac{\sigma}{ne}$ is the effective mobility, $C_{ox}$ is the gate oxide capacitance, $V_{gs}$ is the gate-source voltage and $V_{th}$ is the threshold voltage. According to the formula above, the applied magnetic field enhances the drain current by increasing the conductivity in the channel, which results in negative magnetoresistance.

**Supplementary Note 2. Positive magnetoresistance in silicon nanowire transistors based on Zener tunneling.**

The detailed deductions are demonstrated as follows:

According to the Hall effect, the difference in carrier concentrations between the two sides of the channel is $\Delta n=\frac{2\varepsilon_{o}\varepsilon_{r}}{ed}vB=\beta B$, where $\varepsilon_{o}$ is the vacuum permittivity, $\varepsilon_{r}$ is the relative permittivity, $d$ is the width of the channel, $v$ the carrier velocity and $\beta$ is the adjustable parameter. Under the assumption of the trapezoidal distribution in the space charge region (Fig. 4d), the widths of the space charge region in the channel and the drain are $W_{a}=W_{a0}\left[ a_{a}\left( x+b_{a} \right)+1 \right]$ and $W_{d}=W_{d0}\left[ a_{d}\left( x+b_{d} \right)+1 \right]$, where $W_{a0}$ and $W_{d0}$ are the original widths of the space charge region, *a* and *b* are adjustable parameters and *x* is the location along an interface of the p-n junction, as shown in Fig. 4d.

In addition, according to the WKB approximation ^S2^, $E_{g}+q\Delta V_{d}=qE\cdot W$, where *E_g_* is the band gap width, $W$ is the width of the space charge region and is directly related to the tunneling electric field and $\Delta V_{d}$ is the tunneling window.

The Zener tunneling expression is given by $J_{t}=\frac{q^{2}E}{36\pi\hbar^{2}}\sqrt{\frac{2m^{*}}{E_{g}}}D\cdot\mathrm{Exp}\left( -\frac{4\sqrt{2m^{*}{E_{g}}^{3}}}{3q\hbar E} \right)$, where *E* is the tunneling electric field, $m^{*}$ is the effective mass and *D* the integral ($D=\int\left[ F_{C}\left( E \right)-F_{V}(E) \right]\left[ 1-\exp\left( {-2E_{S}}/\bar{E} \right) \right]dE$). Therefore, the ratio of the tunneling currents with a magnetic field to the original tunneling currents can be calculated as follows,

$\begin{matrix} \frac{I_{B}}{I_{0}}=\frac{1}{L\cdot\text{Exp(X}_{1}\text{)}}\int_{0}^{L} \frac{Exp\left[\frac{\text{X}_{1}}{W_{a0}+W_{d0}}W_{a}+W_{d}\right]}{W_{a}+W_{d}}dx \\ \text{=}\left.\frac{1}{L\cdot\left(a_{1}W_{a0}+a_{2}W_{d0}\right)\cdot\text{Exp(X}_{1}\text{)}}Ei\left[\frac{\text{X}_{1}}{W_{a0}+W_{d0}}W_{a}+W_{d}\right]\right|_{0}^{L} \end{matrix}$,

where , $Ei\left( x \right)$ is the exponential integral function and *L* is the diameter of the silicon nanowire.

**Supplementary References**

1. Altshuler, B. L., Khmel'Nitzkii, D., Larkin, A. I., & Lee, P. A. Magnetoresistance and Hall effect in a disordered two-dimensional electron gas. *Phys. Rev. B*. **22**, 5142 (1980).
2. Sze, S. M., & Ng, K. K. *Physics of Semiconductor Devices*. (John wiley & sons, 2006).
